# A Universal Vector for High-Efficiency Multi-Fragment Recombineering of BACs and Knock-In Constructs

**DOI:** 10.1371/journal.pone.0062054

**Published:** 2013-04-29

**Authors:** Karamjit Singh Dolt, Melanie L. Lawrence, Eve Miller-Hodges, Joan Slight, Anna Thornburn, Paul S. Devenney, Peter Hohenstein

**Affiliations:** 1 MRC Human Genetics Unit, MRC Institute of Genetics and Molecular Medicine, University of Edinburgh, Western General Hospital, Edinburgh, United Kingdom; 2 The Roslin Institute, University of Edinburgh, Midlothian, United Kingdom; Johns Hopkins School of Medicine, United States of America

## Abstract

There is an increasing need for more efficient generation of transgenic constructs. Here we present a universal multi-site Gateway vector for use in recombineering reactions. Using transgenic mouse models, we show its use for the generation of BAC transgenics and targeting vectors. The modular nature of the vector allows for rapid modification of constructs to generate different versions of the same construct. As such it will help streamline the generation of series of related transgenic models.

## Introduction

The generation of transgenic animals has become a central aspect of many biological, biomedical and agricultural projects. There are numerous examples of transgenic models currently being used in research, including ectopic overexpression, site-specific recombination (mainly Cre and CreERT2), reporter models as well as knock-in and knock-out lines. Using these types of models, virtually any question can now be answered *in vivo* in model organisms such as the mouse. However, this limitless potential makes the requirements for model design and generation increasingly strenuous. For instance, whereas conditional knockout experiments were previously achieved using simple Cre alleles, it is now becoming increasingly common to use a Cre driver for one project, a CreERT2 driver for the next, and even GFP-Cre and/or GFP-CreERT2 alleles which combine both reporter and recombinase activity in a single construct (see for instance [1]). There is little doubt that additional variants, for instance fusion constructs with other fluorescent reporters, 2A-based fusion constructs [2] or newly developed site-specific recombinase systems [3], [4] will further increase the need for more flexible cloning systems to generate overexpression or targeting vectors.

It has been shown by many studies that large genomic constructs like YACs, BACs and PACs provide the best option for physiologically-relevant expression of constructs [5]. The development of ‘recombineering’, efficient recombination-based cloning methods in *E. coli* [6], has made BAC vectors the optimal tool for the creation of transgenic models due to a combination of size and ease of manipulation. Moreover, if needed, parts from a (recombineered) BAC can be sub-cloned via a gap repair approach into a normal plasmid backbone for targeting of the corresponding locus [6]. Finally, BAC recombineering can be adapted to high-throughput pipelines for the generation of BAC transgenes and targeting vectors [7], [8]. Thus, improving the efficiency of making variant BAC constructs would have an impact on mouse model generation beyond BAC transgenics themselves.

Here we describe a universal BAC recombineering vector that utilizes Multisite Gateway® cloning technology [9]. The vector consists of Gateway entry cassettes followed by a combined prokaryotic and eukaryotic positive selectable marker. Up to 4 fragments consisting of ORFs, pA sites, IRES sequences or other functional elements can be inserted into or removed from this vector in a single reaction. Individual fragments can easily be replaced by other fragments in a modular fashion. We demonstrate the generation of specific BAC mini-targeting vectors, the efficient replacement of individual fragments and the use of these constructs as BAC transgenic vectors or as a basis for knock-in vectors.

## Materials and Methods

### Multisite Gateway Constructs

All Gateway reactions were done as described in the manual of the MultiSite Gateway® Pro Plus kit (Invitrogen). Gateway fragments were PCR amplified, cloned in the appropriate pDONR vector via a BP reaction and sequence verified. eGFP-Cre and eGFP-CreERT2 template vectors were a kind gift from Dr. Akio Kobayashi (Harvard Medical School). All primer sequences used for amplification and pDNOR vectors are given in table 1. We performed a four-fragment LR^+^ reaction with the pENTR eGFPCRE L1-R5, pENTR IRES L5-L4, pENTR puroR R4-R3, pENTR pA L3-L2 and pcDNA6.2/V5-pL-DEST vectors to make pcDNA6.2-GFPCre-IRES-puroR-pA. The combination of the four fragments was sub-cloned as an attB1/attB2 fragment into pDONR/Zeo to make pENTR/Zeo-GFPCre-IRES-puroR-pA via a reverse BP reaction. We cloned a Gateway ENTR cassette (Gateway® vector conversion system, Invitrogen) into the *Eco*RV site of PL451 [10] and sub-cloned the attB1/attB2 fragment from pENTR/Zeo-GFPCre-IRES-puroR-pA into this via a standard LR reaction. The resulting vector was designated pMULTIrec. All vectors and plasmids in this work can be obtained from Addgene or by request from the corresponding author.

**Table 1:** Primers and pDONR vectors used in the generation of pENTR vectors.

Fragment	Forward	Reverse	pDONR	Result
eGFPCre	GGGGACAAGTTTGTACAAAAAAGCAG GCTTAACCATGGTGAGCAAGGGCGAG	GGGGACAACTTTTGTATACAAAGTTG TCTAATCGCCATCTTCCAG	pDONRP1-P5r	pENTReGFPCre L1-R5
mKate2	GGGGACAAGTTTGTACAAAAAAGCAG GCTTAATGGTGAGCGAGCTGATTA	GGGGACAACTTTTGTATACAAAGTTG TCTATCTGTGCCCCAGTTTG	pDONRP1-P5r	pENTRmKate2 L1-R5
eGFP-CreERT2	GGGGACAAGTTTGTACAAAAAAGCAG GCTTAACCATGGTGAGCAAGGGCGAG	GGGGACAACTTTTGTATACAAAGTTG TTCAAGCTGTGGCAGGGAA	pDONRP1-P5r	pENTReGFPCreERT2 L1-R5
mCherry	GGGGACAAGTTTGTACAAAAAAGCAG GCTTAATGGTGAGCAAGGGCGAG	GGGGACAACTTTTGTATACAAAGTTG TCTACTTGTACAGCTCGTCCATG	pDONRP1-P5r	pENTRmCherry L1-R5
IRES	GGGGACAACTTTGTATACAAAAGTTG TGGTTATTTTCCACCATATTGCCGTC	GGGGACAACTTTGTATAGAAAAGTTG GGTGTATTATCATCGTGTTTTT	pDONRP5-P4	pENTR IRESL5-L4
puroR	GGGGACAACTTTTCTATACAAAGTTG CTACCATGACCGAGTACAAGCCC	GGGGACAACTTTATTATACAAAGTTG TTCAGGCACCGGGCTTGCG	pDONRP4r-P3r	pENTR puroRR4-R3
pA	GGGGACAACTTTGTATAATAAAGTTG CTCTGTGCCTTCTAGTTGCCAGCC	GGGGACCACTTTGTACAAGAAAGCTG GGTAATAGAGCCCACCGCATCC	pDONRP3-P2	pENTR pAL3-L2

### Recombineering

All recombineering reactions were done using the pSim17 plasmid [11] as described elsewhere [12]. The TK counter selection cassette was introduced via recombineering with PL611 as described [11]. BAC constructs used for microinjection were subjected to an additional recombineering step to remove the loxP site from the vector backbone. An Amp^R^ selection cassette was amplified from pRosa26-DEST [13] using forward primer gcttatcgatgataagctgtcaaacatgagaattgatccggaacccttaatcttacaatttaggtggcact and reverse primer tccgatgcaagtgtgtcgctgtcgacggtgaccctatagtcgagggacctaatatgagtaaacttggtctga and recombineered into the BAC as described [12]. Correct integration in the BAC backbone was confirmed using PCR (LoxPtoAmptestF: gtgccgaggatgacgatgagc; LoxPtoAmptestR: ccgtgccggcacgttaacc).

### BAC Microinjections

BAC constructs were isolated using the Large Construct kit (Qiagen). They were resuspended in BAC injection buffer (10 mM TRIS-HCl pH 7.5; 0.1 µM EDTA; 100 mM NaCl; 70 µM spermidine; 30 µM polyamines (freshly made) and injected in F1 CBA/C57BL/6J E0.5 fertilized oocytes at a concentration of 0.5 or 1 ng/µl using a Femtotip injection needle (Eppendorf) with an inner diameter of 0.2 um. Injected embryos were cultured overnight and transferred to pseudo-pregnant CD1 females. BACs used were bMQ317G18 (*Nanog*) and RPCI23-311C1 (*Six2*).

### ES Cell Targeting

The retrieved Six2-GCiP targeting construct was linearized with *Not*I and electroporated into G4 ES cells [14] as described previously [15]. Clones were screened for correct homologous recombination by long range PCR using 3′ external primers and a nested PCR (F: TCGACTAGAGCTTGCGGAACC; R: ATGACTCGGAAGACACAGGCTACT; F_nested_: CAAATAGTATAGGAACTCATGGTC; R_nested_: GCAGAAGTGTGGGCTATG). Southern blot was performed to analyse the correct integration at the 5′ end in the clones selected by long range PCR. Genomic DNA was digested with *Eco*RI. A 500 nt PCR fragment (F: AGCACCGAAGTTGTATCTG; R: AATGCTGCTCCTTCTGTC) was used as probe. A correct targeted clone was used for the generation of mice using the diploid morula aggregation method [16].

### iPS Reprogramming

iPS cells were generated using a piggyBac system expressing Oct4, Sox2, Klf4, c-Myc and Lin28 from a single vector as a 2A-linked polypeptide as described [17].

### Mouse Models

All mouse models were genotyped via PCR with the following primers. *Six2*-GCiP BAC and *Six2* ^+/GCiP^ knock-in (Cre/F: GCATTACCGGTCGATGCAACGAGTGATGAG; Cre/R: GAGTGAACGAACCTGGTCGAAATCAGTGCG); *Nanog*-KiP BAC (mKate2/F: TGACCGCTACCCAGGACACCA; mKate2/R: GACGCCGGGCATCTTGAGG); *Rosa26* ^tdRFP^ (Gt(ROSA)26Sortm1Hjf) [18] (R26g2/F: TGTTATCAGTAAGGGAGCT; R26g2/Rmut: TGTTATCAGTAAGGGAGCT; R26g2/Rwt: CACACCAGGTTAGCCTTTA).

### Kidney Organ Culture and Time-lapse Imaging

Embryonic kidneys from *Six2* ^+/GCiP^ x *Rosa26* ^+/tdRFP^ crosses were micro-dissected and were cultured on Transwell Multiwell polyester membrane 0.40 µm pore size inserts in six-well plates (Corning) in 10% Serum DMEM and left in a fully humidified 37°C incubator with 5% CO_2_ for an hour until the kidneys had attached to the membranes and the epithelial structures were flat enough for the microscope to be focused accurately. Kidneys were scanned every half hour for the 2 days’ period of culture using a Nikon TiE (Perfect Focus System) microscope with NIS-Elements 4.0 equipped with an incubation chamber at 37°C, 100% humidity, and 5% CO2. Cultures were started from E13.5 kidneys and followed for 48 hours. Antibody staining was done using primary anti-Cdh1 monoclonal antibodies (610182, BD Transduction laboratories) and secondary Alexa Fluor®647 Donkey-anti-Mouse IgG (A31571, Life Technologies).

#### Ethics statement

All animal work was approved by the ethical review committee of the University of Edinburgh and according to UK home office legislation (project license PPL 60/3788).

## Results

**Figure 1:**
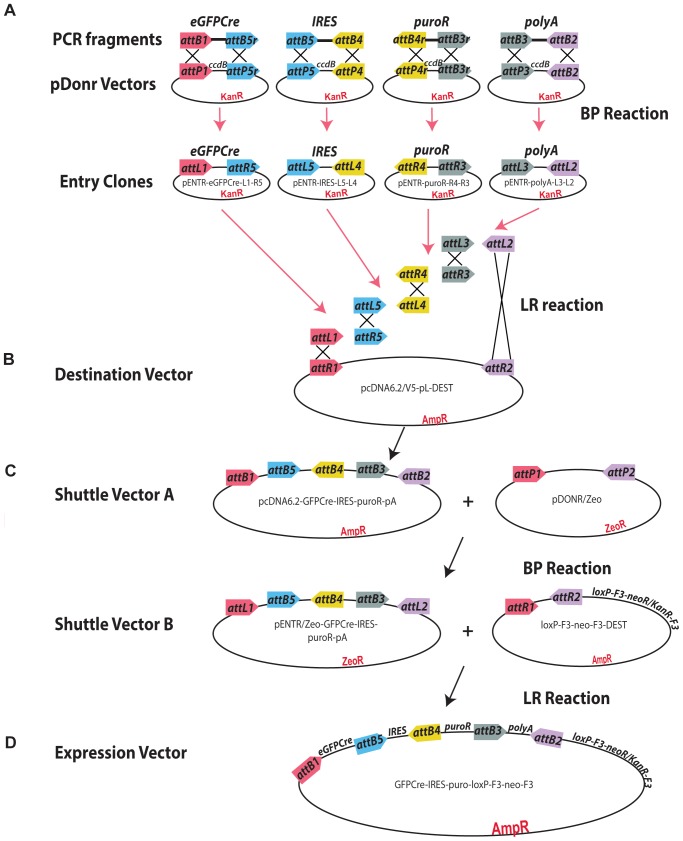
Generation of pMULTIrec. **A.** Four multisite Gateway compatible fragments (eGFPCre; IRES; puro^R^ and pA) were PCR amplified and cloned into the appropriate pDONR vector to make four pENTR vectors. **B.** The four pENTR clones were combined into one four-fragment Gateway clone. **C.** The four-fragment cassette was moved to pDONR/Zeo to change antibiotic resistance and subsequently sub-cloned to a vector carrying a Gateway ENTR cassette upstream of a dual selection (Neo/Kan) cassette. **D.** The resulting pMULTIrec vector.

**Figure 2:**
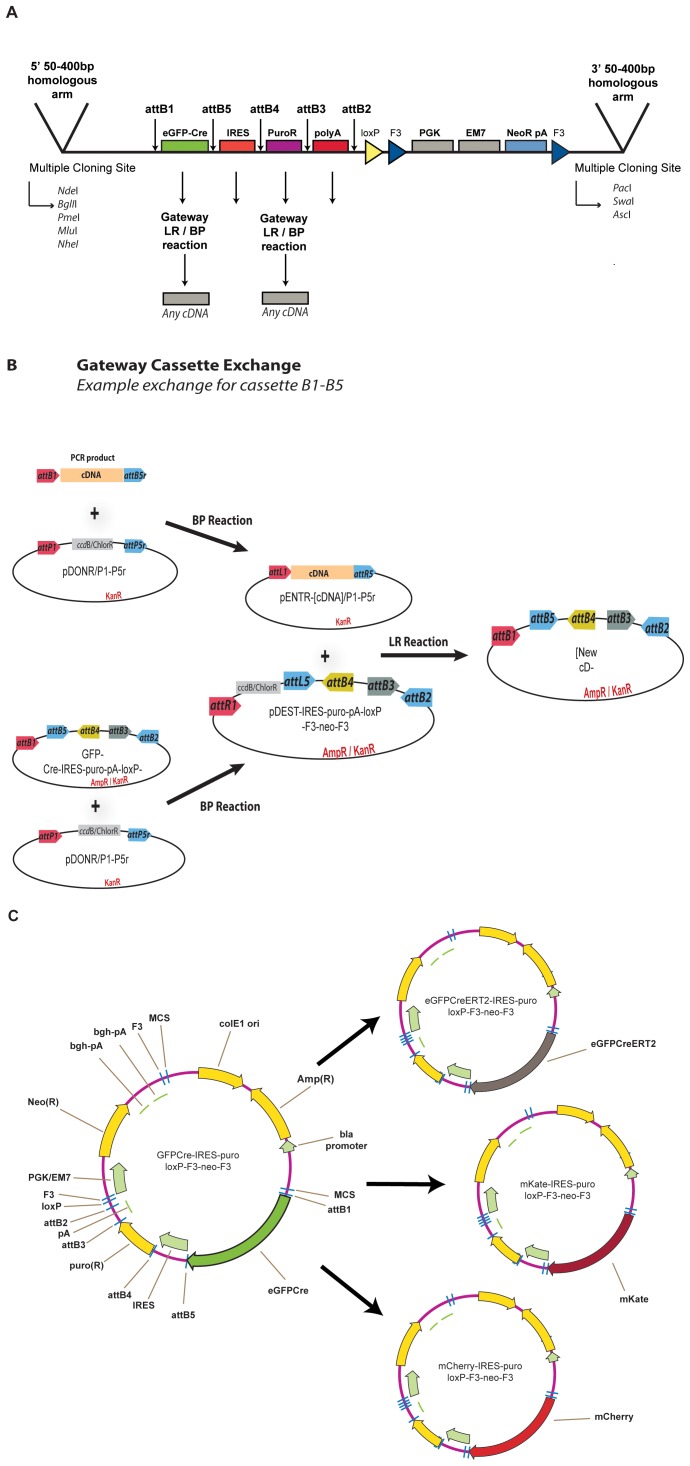
Additional flexibility in pMULTIrec. **A.** Detailed schematic (not to scale) of *att* configuration and multiple cloning sites in pMULTIrec. **B.** Example of Gateway cassette exchange. Any cDNA of interest can be PCR-amplified cloned into the desired pDONR vector to generate a new pENTR clone. The same pDONR vector is used in a BP reaction with pMULTIrec, transformed in *ccdB* ^R^ bacteria and selected using Amp/Cam double selection. This generates a new DEST vector with the ENTR cassette in place of the desired fragment. This new cDNA is transferred to this new DEST vector via a standard LR reaction. **C.** Additional pMULTIrec variants we have generated.

As a basis for our modular recombineering construct (pMULTIrec) we generated a 4- fragment construct (eGFPCre-IRES-puromycin^R^-pA) upstream of a combined prokaryotic/eukaryotic selection cassette (Figure 1). Gateway fragments were PCR amplified, cloned into pDONR vectors and sequence verified (data not shown). Several sub-cloning steps were necessary due to antibiotic resistance incompatibility of the vectors. The resulting vector contains 5′ and 3′ multiple cloning sites to allow cloning of homologous arms for a recombineering reaction. Via recombineering, this construct can be targeted to the start codon of any gene of interest on a BAC to express an eGFPCre fusion protein under the control of the BAC gene promoter as well as puromycin resistance from the locus of interest. Additional flexibility of the construct can be obtained through reverse Gateway reactions (Figure 2).

Each of the four Gateway cassettes is flanked by a different combination of specific *att* sites (Figure 2a). Therefore, a BP reaction with any of the multi-fragment pDONR clones will result in the replacement of only the corresponding fragment by a new ENTR cassette; this can subsequently be replaced by a new functional element through a standard LR reaction (Figure 2b). In this way we could rapidly generate versions of the vector to express eGFP- CreERT2, mKate2 (far-red fluorescent protein) and mCherry together with the puromycin resistance gene (Figure 2c; Table 2). Other fragments can be exchanged the same way.

**Table 2:** Efficiency of the Gateway reactions used.

Constructs	Gateway reaction	Colonies tested	Correct Digest pattern	Resulting construct
pMULTIrec+pDONR P1-P5r	BP	8	5	pDEST-IRES-puro-pA
pENTR mKate2 L1-R5+ pDEST-IRES-puro-pA	LR	12	5	mKate2-IRES-puro-pA
pENTR eGFPCreERT2 L1-R5+ pDEST-IRES-puro-pA	LR	12	5	eGFPCreERT2-IRES-puro-pA
pENTR mCherry L1-R5+ pDEST-IRES-puro-pA	LR	3	3	mCherry-IRES-puro-pA

**Figure 3:**
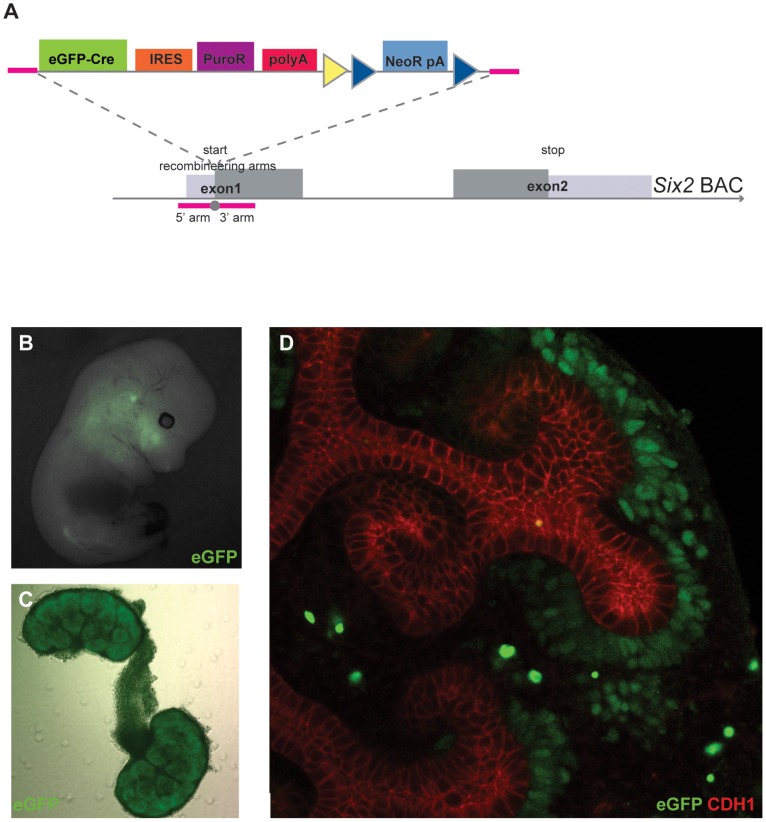
The *Six2*-GCiP BAC construct made using pMULTIrec. **A.** The four-fragment and dual selection cassette from pMULTIrec was cloned in a *Six2* containing BAC replacing the start codon. **B.** GFP imaging of a E13.5 embryo carrying the *Six2*-GCiP BAC. **C.** GFP imaging of whole mount E13.5 kidneys. **D.** E13.5 *Six2*-GCiP BAC kidneys cultured for four days showing GFP expression in the cap mesenchyme and Cdh1 expression in the ureteric bud.

Kidney development is a dynamic process that starts with bidirectional communication between the mesenchymal cells from the metanephric mesenchyme and the epithelial cells from the invading ureteric bud; this results in the mesenchymal cells undergoing a mesenchyme-to-epithelial transition to form the nephrons, the filtering units in the adult kidney [19]. Expression of the *Six2* gene labels the nephrogenic progenitor cells in the developing kidney, as was shown using extensive lineage tracing and based on several different targeting constructs for the same locus [1]. This provided us with a useful target locus to test our system. We first inserted 5′ and 3′ homologous arms to allow us to target the eGFPCre-IRES-puromycin^R^-pA recombineering vector (hereafter referred to as GCiP) to the start codon of the *Six2* locus. The resulting construct was recombineered into a BAC carrying the complete *Six2* locus (Figure 3a; Table 3) and was microinjected into oocytes to generate transgenic mice. The GFP expression pattern of embryos positive for the *Six2*-GCiP BAC (Figure 3b) was identical to the pattern described before [1], indicating no ectopic expression domains resulted from any site or copy number effects. Within the developing kidney GFP positive cells were found in the cap mesenchymal cells lining the ureteric bud (Figure 3c,d), the known location of the Six2-positive progenitor cells [1]. We therefore concluded that our universal BAC recombineering vector can faithfully express inserts via BAC transgenics.

**Table 3:** Efficiency of the recombineering reactions used.

Constructs	Colonies tested	Correct 5′ and 3′ PCRbased test for integration	Desired construct
Six2-eGFPCre-IRES-puro-pA+Six2-BAC	10	10	Six2-eGFPCre-IRES-puro-pA BAC
Nanog-mKate2-IRES-puro-pA+Nanog-BAC	6	6	Nanog-mKate2-IRES-puro-pA BAC

**Figure 4:**
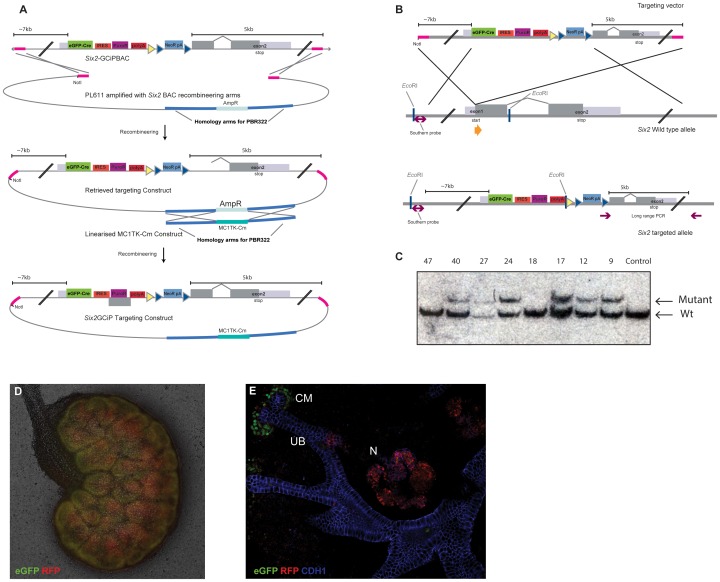
The *Six2* ^+/GCiP^ allele based on the pMULTIrec system. **A.** Retrival of the *Six2*-GCIP targeting vector. **B.** Targeting of the *Six2* locus. **C.** Confirmation of correct targeting of the *Six2* locus (restriction enzyme and probe indicated in figure 4B). **D.** *Six2* ^+/GCiP^ *Rosa26* ^tdRFP^ kidney in culture showing GFP and RFP signals. **E.** *Six2* ^+/GCiP^ *Rosa26* ^tdRFP^ kidney in culture showing GFP and RFP signals and Cdh1 antibody staining. CM: cap mesenchyme; UB: ureteric bud; N: nephron.

To determine if our universal construct allows expression from endogenous loci, we retrieved a targeting construct with 7 and 5 kb homologous arms from the *Six2*-GCiP BAC into a plasmid backbone, and added a TK counter selection gene through a second recombineering reaction (Figure 4a). This vector was used to target the endogenous *Six2* locus in mouse embryonic stem (ES) cells (Figure 4b). A combined long-range PCR and Southern blot approach was used to identify correctly targeted clones. We tested 95 G418+ gancyclovir resistant clones via long-range PCR to detect correct integration at the 3′ end of the targeting construct, which yielded 22 candidate clones (data not shown). Eight of these candidate clones were tested via Southern blot screening, 6 of which were confirmed to be correctly targeted (Figure 4c), which we used to _generate *Six2*_ *^+/^* ^GCiP^_ knock-in mice. As shown for previous *Six2* knockout and_ knock-in models [1], [20], heterozygous *Six2* ^+/GCiP^ mice were viable, healthy and fertile (data not shown).

The *Six2* ^+/GCiP^ allele was tested in kidney organ culture after crossing to a *Rosa26* ^tdRFP^ Cre reporter allele [18] to follow the lineage of the *Six2* positive cells and test the Cre moiety of the construct. We used time-lapse imaging of the brightfield, GFP and tdRFP signals to illustrate the dynamics of this lineage trace (Figure 4e and Movie S1). In accordance with the role of Six2-positive cells as nephrogenic progenitor cells, green cells (GFP-positive only) were found in the cap mesenchyme surrounding the ureteric bud. These cells rapidly became RFP-positive while remaining GFP-positive, due to the Cre-mediated activation of the tdRFP reporter allele. As Six2-positive cells go through the MET during nephron development they switch off *Six2* expression [21], [22], and as a result the post-MET nephrons on the *Six2* ^+/GCiP^ *Rosa26* ^+/tdRFP^ kidneys were GFP-negative but remained RFP- positive (Figure 4f and Movie S1). These data confirm that our universal recombineering vector can be used for the generation of targeting vectors as well as the generation of transgenic BAC models.

**Figure 5:**
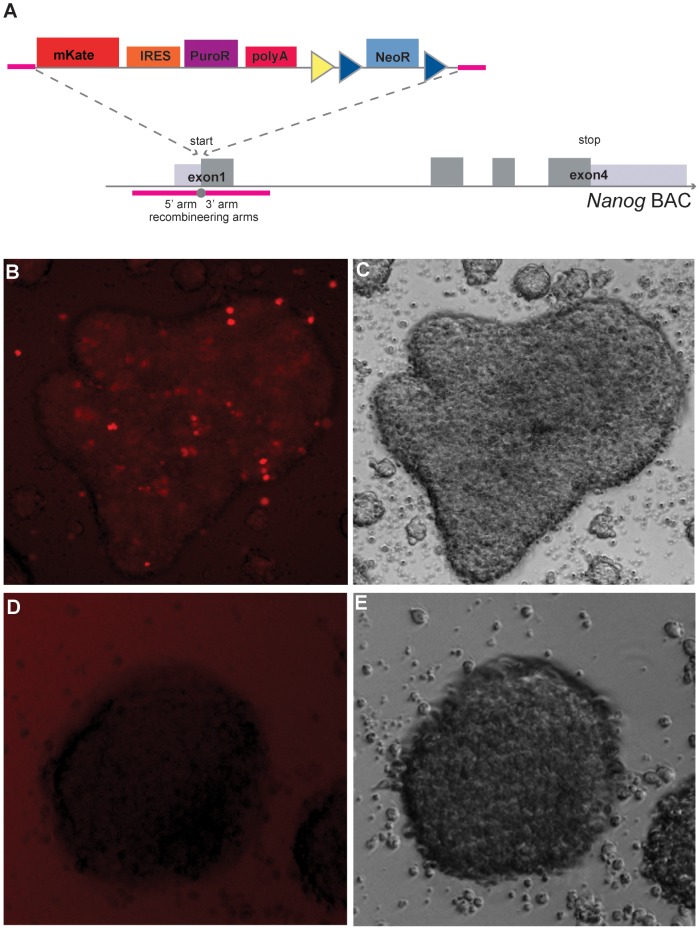
The *Nanog*-KiP BAC made from a modified pMULTIrec vector. **A.** Generation of the *Nanog*-KiP BAC. **B, C.** mKate2 (B) and brightfield signals of iPS clones derived from *Nanog*-KiP BAC MEFs. **D, E.** mKate2 (B) and brightfield signals of iPS clones derived from wild type MEFs.

To further illustrate the flexibility of our vector, we generated a mouse model that expresses the mKate2-IRES-puromycin^R^-pA (KiP) cassette from the *Nanog* locus using a BAC construct (Figure 5a). *Nanog* is an essential pluripotency gene in embryonic stem cells, and its activation is an important marker for full reprogramming of somatic cells into induced pluripotent stem (iPS) cells [23]. MEFs from the Nanog-KiP mouse model, as well as MEFs from wild type littermates, were used to generate iPS cells using a piggyBac-based reprogramming system [17]. Whereas MEFs from the wild type did not show any fluorescence, the *Nanog*-KiP BAC iPS cells showed a clear signal in the far-red channel (Figure 5b–e). We observed some degree of intercellular variation in the signal, which could be the result of the intraclonal fluctuations in *Nanog* expression [24].

## Discussion

Here we describe a universal, high-efficiency vector, pMULTIrec, for the generation of multi-fragment expression constructs that is compatible with Gateway® cloning. Starting from a four-fragment construct, any of the inserts can efficiently be replaced by another fragment using a two-step Gateway reaction. Although so far we have only generated four- fragment constructs, using the commercially available MultiSite Gateway Pro Plus® system (Invitrogen) it will be possible to make 1, 2 and 3-fragment constructs as well, providing a truly universal vector system.

Our system has several features that make it a flexible and efficient starting point for the generation of transgenic animal models. First, through implementation of the MultiSite Gateway cloning system different variations of transgenic constructs can easily be generated. Here we demonstrate this flexibility by making variations of BAC targeting vectors, and modifying an existing targeting construct that was retrieved from a BAC recombineered with this construct should be equally efficient. In theory it should be possible to remodify recombineered BAC constructs generated with the pMULTIrec system; however this would require *ccdB* ^R^ bacteria suitable for maintaining BACs which to our knowledge are not available. Second, the 5′ and 3′ multiple cloning sites allow easy insertion of short homologous arms for use in the BAC recombineering steps. Here we have generated these arms through PCR using the target BAC as template. However, as prices for commercial gene synthesis services are continuously decreasing, this might be a viable option as well. Third, after integration of the construct into the mouse genome, FLP recombinase can be used to remove the dual selection cassette allowing it to be reused in another construct.

Other ways of generating recombineering cassettes include PCR approaches and DNA synthesis. However, vectors resulting from any PCR-based method must be sequence-verified for every construct or project. This is the case with commercially available systems, like In-Fusion® (Clontech) and non-commercial systems, like SLIC [25] and SLiCE [26]. Synthesis of DNA fragments of the sizes needed for the generation of complete expression cassettes is still financially challenging for many laboratories, and difficult sequences to synthesize, like high GC or repetitive sequences, will add additional costs and delays. The power of the pMULTIrec system over these alternatives lies in its modular nature. Once a fragment has been generated and sequence-verified, it can be re-used in unlimited configurations.

So far we have generated 4 different versions of the vector, each with a varying first fragment, and targeted them to four different BACs and/or endogenous genes. In our experience a variation of the pMULTIrec vector can be generated in a few days once the fragment is available in the appropriate Gateway ENTR clone. Our constructs have been knock-in constructs at the start codon of the gene of interest. However, with the correct design of the 5′ homologous arm it should also be possible to use our vector for C-terminal tagging of genes. In addition, although all our work has been focused on the mouse as experimental system, the vector presented here can just as easily be used for other model organisms for which ES cell- or BAC-mediated transgenesis is possible. Recent developments in the use of Zinc Finger Nuclease- and TAL Effector Nuclease-assisted homologous recombination with short homologous arms in many different cell types from different species [27], [28] could further extend the use of the vector described here; the same short homologous arms that are used here to target a BAC construct might be sufficient to target endogenous loci with the help of engineered nucleases. This would make the system described here a truly universal one.

## Supporting Information

Movie S1 **Time-lapse movie of 2-day culture of E13.5** ***Six2*** **^+/GCiP^** ***Rosa26*** **^tdRFP^ kidneys.**
(MOV)
